# ARIH1 inhibits influenza A virus replication and facilitates RIG-I dependent immune signaling by interacting with SQSTM1/p62

**DOI:** 10.1186/s12985-023-02022-1

**Published:** 2023-04-01

**Authors:** Shengyu Wang, Zhenrong Li, Yaping Chen, Sanli Gao, Junhua Qiao, Haoru Liu, Hong Song, Dishu Ao, Xin Sun

**Affiliations:** 1 Key Laboratory of Infectious Disease and Biosafety, Provincial Department of Education, Guizhou, Institute of Life Sciences/ College of Preclinical Medicine, Zunyi Medical University, Zunyi, China; 2 Department of Microbiology, College of Preclinical Medicine, Zunyi Medical University, Zunyi, China

**Keywords:** ARIH1, RIG-I, SQSTM1, p62, Innate immunity, Influenza A virus

## Abstract

**Background:**

Multiple host factors are involved in modulating type I interferon expression induced by viruses; however, the mechanism is not fully elucidated. Influenza A virus infection causes severe respiratory symptoms and triggers a series of signaling cascades and host innate immune responses, including interferon production. The co-IP/MS technology was used to screen several antiviral factors in the early stage. Among these factors, ariadne-1 homolog (ARIH1) caught our attention.

**Methods:**

Western blot assay was performed to detect the level of proteins and software ImageJ was used to analyze the band intensities. Polymerase activity assay was conducted to evaluate the polymerase activity of influenza A virus. Tissue culture infective dose (TCID_50_) assay was performed to measure influenza A virus titers, and quantitative RT-PCR assay was applied to test the mRNA level of IFN-β, ISG56, and CXCL10. Luciferase reporter assay was used to confirm the target of ARIH1 in RIG-I signaling. Immunoprecipitation assay was performed to detect the interaction and the ubiquitination of the proteins. All data were analyzed by biostatistical methods and presented as means ± standard deviation from three independent experiments. Statistical significance was determined using two-tailed student’s *t* test. A *P* value of less than 0.05 was considered statistically significant, and a *P* value of less than 0.01 was considered highly significant (ns, *P* ≥ 0.05; *, *P* < 0.05; and **, *P* < 0.01).

**Results:**

We found that ARIH1, a member of E3 ubiquitin ligases, enhanced cellular antiviral responses. Subsequent study showed that ARIH1 was up-regulated during influenza A virus infection. Further analysis showed that ARIH1 enhanced IFN-β and downstream gene expression by affecting the degradation of RIG-I through the SQSTM1/p62 signaling pathway.

**Conclusion:**

This newly revealed mechanism shows that cellular response increases of ARIH1 and promotes IFN-β expression to boost host survival during viral infection.

**Supplementary Information:**

The online version contains supplementary material available at 10.1186/s12985-023-02022-1.

## Introduction

Host immune responses are divided into innate and adaptive immune responses, with the former acting as the first line of defense against infection [1–3]. During virus infection, the conserved components called pathogen associated molecular patterns are recognized by host pathogen recognition receptors, leading to the activation of innate immune signaling and production of a series of cytokines and inflammatory factors [4, 5]. The induction and action of type I interferon are important in human immune defense activation and intracellular antimicrobial progress by influencing the development of innate and adaptive immune responses, up-regulating antiviral responses, and restricting viruses replication [6, 7]. Influenza A virus is a single-strand negative-sense RNA virus contagious pathogen that is responsible for severe respiratory infection in humans and animals worldwide and has received attention in the current COVID-19 pandemic [3, 8]. The virus also induces type I interferon production primarily through the activation of RIG-I pathway, during which multiple host factors are involved [1, 9–12].

Retinoic acid-inducible gene-I (RIG-I) is an important member of RIG-I-like receptors that detect viral nucleic acids in the cytosol [13–15]. Once activated, RIG-I triggers the expression of downstream MAVS, TRAF3, and TBK-1/IKKε and ultimately induces IRF3 phosphorylation, nuclear localization, and type I interferon expression [5, 16]. The expression of RIG-I and its polyubiquitination of N-terminal CARD domain (RIG-I-N) are critical for its function and are regulated by a variety of host factors [13, 15, 17].

Ariadne-1 homolog (ARIH1) is a member of the Ariadne family of E3 ubiquitin ligases with many biological functions. ARIH1 is involved in tumorigenesis, cell development, and metabolism; however, its role in antiviral innate immunity is not fully known [18, 19]. In this study, we found that influenza A virus, wild-type H1N1 virus A/ PR8 (H1N1/PR8), could up-regulate the expression of ARIH1 as a novel positive regulator of RIG-I signaling. ARIH1 also promotes the RIG-I induced production of type I interferon by interacting with SQSTM1/p62. Our findings reveal a new host factor of defense influenza A virus evasion by regulating antiviral immune response.

## Materials and methods

### Cell culture and transfection

Human type II alveolar epithelial (A549) cells cultured in Ham’s F12K medium (F-12, BasalMedia, China) with 10% fetal bovine serum (FBS, Gibco, Brazil) and 5% CO_2_ at 37 °C. Human embryonic kidney (HEK293T) cells cultured in RPMI-1640 (BasalMedia, China) and Madin-Darby canine kidney (MDCK) cells were maintained in Dulbecco's minimal essential medium (DMEM, BasalMedia, China) with 10% FBS and 5% CO_2_ at 37 ℃.

For the transient overexpression of specific proteins, cells were transfected with plasmid using Lipofectamine™2000 or Lipofectamine™3000 (Invitrogen). For gene silencing, ARIH1 siRNA (siARIH1) and control siRNA (siNC) were obtained from GenePharma (China). Cells were transfected with siRNAs using Lipofectamine™2000 or Chemi-Trans™ RNAiMAX Transfection Reagent (GeneCodex) following the manufacturer's protocol at a final concentration of 100 nM.

### Antibodies and plasmids

Mouse monoclonal Flag tag antibody was from Sigma (USA); rabbit polyclonal HA tag antibody, rabbit polyclonal ARIH1 antibody and rabbit polyclonal IRF3 antibody were from ABclonal Biotechnology (China); rabbit polyclonal phosphorylated IRF3 (p-IRF3) antibody was from Abcam (USA); mouse monoclonal Myc tag antibody, rabbit polyclonal NP and M1 of H1N1/PR8 antibody and rabbit polyclonal RIG-I antibody were from Proteintech (Wuhan, China); mouse polyclonal GAPDH antibody were from BioPM (Wuhan, China); horseradish peroxidase (HRP) conjugated secondary antibodies were from Beyotime Biotechnology (China).

ARIH1, RIG-I, RIG-I-N (The N-terminal CARD domain of RIG-I), VISA, TBK1, and SQSTM1 expression plasimds; a luciferase reporter plasmid for the IFN-β promoter (pIFN-β-Luc); and a luciferase reporter plasmid pRNP-Luc with a pol I transcription unit were constructed by our laboratory. A Renilla control plasmid (pGL4.75 hRluc/CMV) was purchased from Promega. The PHW-PR8-PB2, PB1, PA, and NP plasmids were constructed from H1N1/PR8 as previous described [20]. Myc-tag ubiquitin plasmid (pMyc-UbWT), Myc-tag ubiquitin mutant in which all lysine residues except K63 were mutated to arginine plasmid (pMyc-UbK63), and Myc-tag mutant in which only the K63 residue was mutated to arginine plasmid (pMyc-UbK63R) were get from Hedgehogbio (Shanghai, China).

### Virus preparation and infection of cells

H1N1/PR8 and Sendai virus (SeV) were grown in 10-day-old fertilized eggs and stored at − 80 °C. The viral titer of H1N1/PR8 was measured using the Reed-Muench method. All H1N1/PR8 experiments were performed in Biosafety Level 2 facilities at the Key Laboratory of Infectious Disease & Biosafety, Provincial Department of Education, Guizhou, Zunyi Medical University, China.

For H1N1/PR8 infection, A549 cells were washed three times in phosphate buffer saline (PBS) to remove FBS and then incubated with the virus diluted in F-12 with 0.5 μg/ml TPCK treated trypsin for 1 h at 37 °C. After 1 h, the cells were washed and maintained in F-12 with 1% FBS, 1% penicillomycin and 0.5 μg/ml TPCK treated trypsin for the indicated times. For IFN-β expression, HEK293T cells and A549 cells were stimulated with SeV.

### Western blot assay

Cells were lysed in RIPA lysis buffer (CST, USA) containing protease inhibitors (Calbiochem) for 30 min on ice and were then centrifuged at 10,000 rpm for 10 min. The supernatant was quantified by Detergent Compatible Bradford Protein Assay Kit (Beyotime Biotechnology, China), added the 1 × SDS Loading buffer and boiled for 5 min. The sample was separated on 10% SDS-PAGE, transferred to nitrocellulose membranes (PALL, Japan), and blocked with 5% bovine serum albumin (BSA), followed by immunoblotting with the indicated antibodies. Immunoreactive bands detected using ECL reagents (Advansta, USA) were developed by Image Lab system (Bio-Rad, USA).

### Polymerase activity assay

HEK293T cells in 12-well plates was transfected with 0.5 μg of PHW-PR8-PB2, PHW-PR8-PB1, PHW-PR8-PA, PHW-PR8-NP, and pRNP-Luc; 0.01 μg of pGL4.75 hRluc/CMV; and 0.5 μg of ARIH1 expression plasmid or control plasmid using 6 μl of Lipofectamine^TM^2000. The cells were incubated for 24 h and then lysed in 200 μl of passive lysis buffer. Luciferase and Renilla activities were assessed using a Dual-Luciferase Assay Kit (Promega).

### TCID_50_ assay

Cell supernatants containing the virus were serially diluted tenfold with DMEM and applied in quadruplicate to 2 × 10^4^ MDCK cells /well in a 96-well plate. On the fifth day post infection, the viral titer was determined by observing the cytopathogenic effect and was confirmed by hemagglutination. The TCID_50_ was determined based on the Reed-Muench method.

### Quantitative RT-PCR assay

Total cellular RNA was extracted using TransZol Up Kit, reversed by TransScript^®^ One-Step gDNA Removal and cDNA Synthesis SuperMix, and analyzed using PerfectStart™ Green qPCR SuperMixt following the instruction. All the reagents were purchased from Transgen Biotech, Beijing, China. All the primers were synthesized from Sangon Biotech, Shanghai, China. The primers used were: 5′-CTCTCCTGTTGTGCTTCTCC-3′ and 5′-GTCAAAGTTCATCCTGTCCTTG-3′ for IFN-β detection; 5′-GCGCTGGGTATGCGATCTC-3′ and 5′-CAGCCTGCCTTAGGGGAAG-3′ for ISG56 detection; 5′-GAAAGCAGTTAGCAAGGAAAGGT-3′ and 5′-GACATATACTCCATGTAGGGAAGTGA-3′ for CXCL10 detection; 5′-GACAACTTTGGTATCGTGGAA-3′ and 5′-CCAGGAAATGAGCTTGACA-3′ for GAPDH detection.

### Luciferase reporter assay

HEK293T cells in 12-well plates were transfected with 0.5 μg of pIFN-β-Luc; 0.01 μg of pGL4.75 hRluc/CMV; 0.5 μg of RIG-I, RIG-I-N, VISA, TBK1, or IRF3; and 2 μg of the ARIH1 expressing plasmid or control plasmid using 6 μl of Lipofectamine™2000. The cells were incubated for 24 h and then lysed in 200 μl of passive lysis buffer. Luciferase and Renilla activities were assessed using a Dual-Luciferase Assay Kit (Promega).

### Immunoprecipitation assay

Cells were lysed in RIPA lysis buffer (CST, USA) containing protease inhibitors (Calbiochem) for 30 min on ice and were then centrifuged at 12,000 rpm for 15 min. The supernatant was incubated with antibody for 1 h at 4 °C and the lysate-antibody complexes were incubated with Protein A/G Magnetic Beads (MedChemExpress) for 6 h at 4 °C. The precipitated agarose was washed four times with lysis buffer to remove nonspecific binding. The immune complex was eluted with 2 × SDS Loading buffer and boiled, separated on SDS-PAGE and analyzed by western blot assay.

### Statistical analysis

The data are presented as means standard deviations (SD) from three independent experiments. Statistical significance was determined using two-tailed Student’s *t* test. A *P* value of less than 0.05 was considered statistically significant, and a *P* value of less than 0.01 was considered highly significant (*, *P* < 0.05 and **, *P* < 0.01). For western blot assay, the band intensities were analyzed using the software ImageJ.

## Results

### ARIH1 impairs influenza A virus replication

**Fig. 1:**
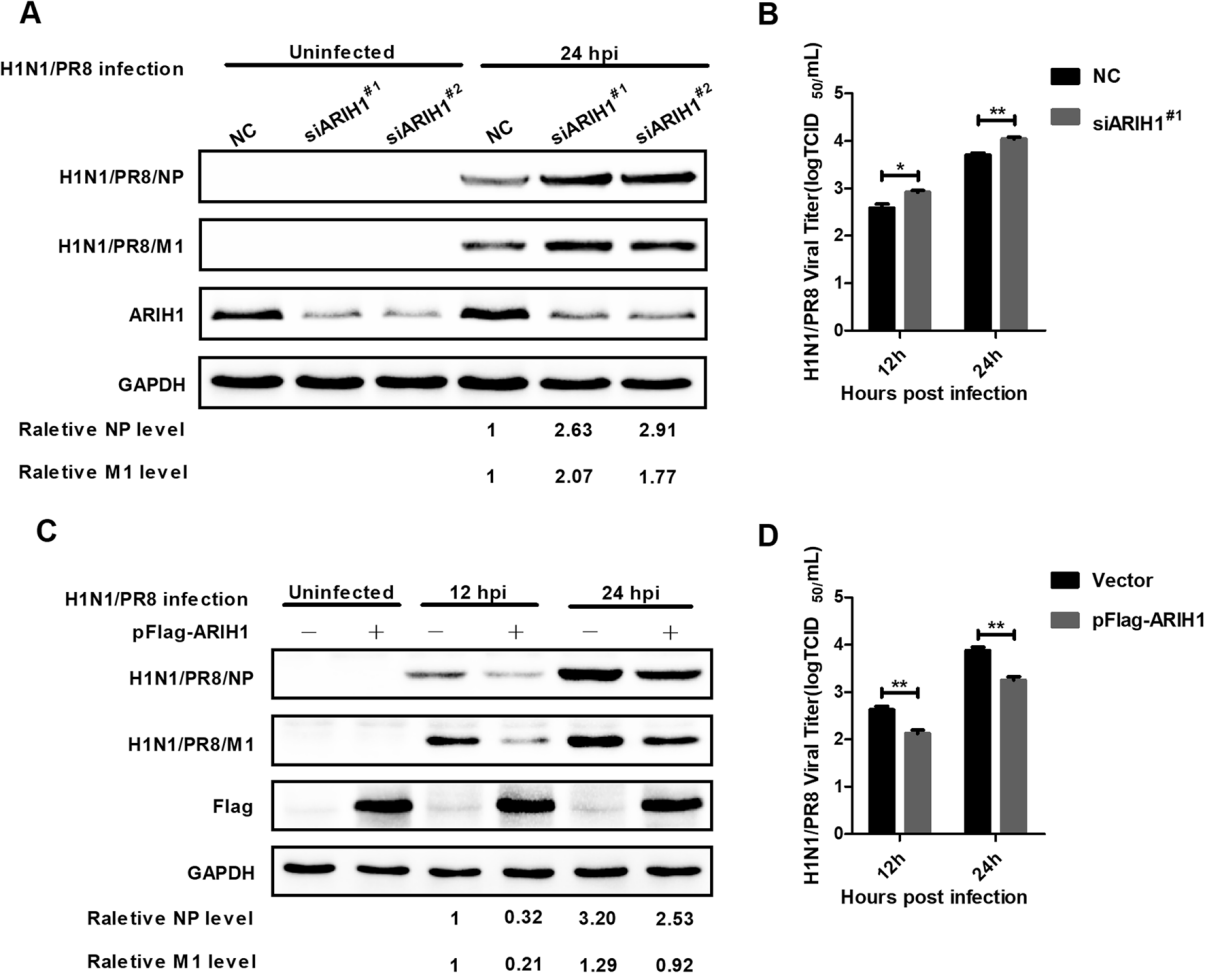
ARIH1 conferred cellular resistance to H1N1/PR8 infection. **A** A549 was transfected with siARIH1^#1^, siARIH1^#2^ or siNC for 36 h, and the relative M1 and NP expression of H1N1/PR8-infected cells at 24 hpi was measured by Western blot assay. **B** TCID_50_ assay was also performed to measure the titer of the culture supernatant of the above cells at 12 and 24 hpi. Data are presented as means ± SD from three independent experiments. *, *P* < 0.05 and **, *P* < 0.01 as determined by student’s *t* test. **C** Plasmid expressing Flag-tag ARIH1(pFlag-ARIH1) or its vector was transfected in A549 cells. After 24 h, the cells were infected with H1N1/PR8 for 12 or 24 h and the cell lysates were analyzed by Western blot assay using antibodies specific for Flag-tag, M1, NP, and GAPDH. **D** Amount of progeny virus in the supernatant from treated cells determined by TCID_50_ assay. Data are presented as means ± SD from three independent experiments. **, *P* < 0.01 as determined by student’s *t* test

Three siRNAs targeting ARIH1 mRNA were synthesized, and their silencing effects were evaluated by Western blot assay and compared with that of scrambled siRNA (siNC) (Additional file 1: Fig. S1). Endogenous ARIH1 was silenced by the two effective siRNAs to investigate the roles of ARIH1 during influenza A virus infection. Western blot analysis revealed that the expression of M1 and NP of the virus was up-regulated at 24 h post-infection (hpi) (Fig. 1A). The viral titers of supernatant collected from ARIH1-knockdown cells at 12 and 24 hpi were determined by TCID_50_ assay and compared with that from the supernatant collected from siNC-treated cells. The results showed that the number of progeny virus in the ARIH1 knockdown cells increased at 12 and 24 hpi (Fig. 1B). These data suggested that ARIH1 down-regulation leads to the efficient replication of H1N1/PR8.

Virus replication was monitored in cells with protein overexpression to confirm whether ARIH1 plays a negative regulatory role during H1N1/PR8 infection. Western blot assay showed that ARIH1 overexpression reduced M1 and NP expression compared with that in a control vector in the A549 cells at 12 and 24 hpi (Fig. 1C). TCID_50_ assay revealed that ARIH1 overexpression reduced the virus titers of cell supernatant compared with that of empty vector at 12 and 24 hpi (Fig. 1D). These results indicated that ARIH1 overexpression suppresses H1N1/PR8 replication.

### ARIH1 is up-regulated upon influenza A virus infection

**Fig. 2:**
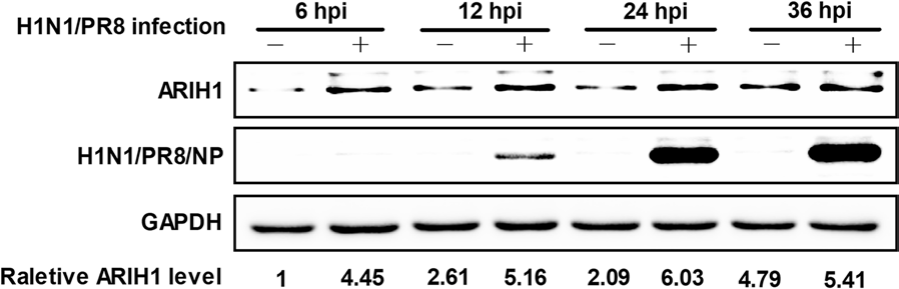
Influenza A virus infection increased endogenous ARIH1. A549 cells were infected with H1N1/PR8 for 6, 12, 24, and 36 h. The cell lysates were examined by Western blot assay using antibodies against ARIH1, NP, and GAPDH

Given that ARIH1 could significantly inhibit the proliferation of influenza A virus, lysates from infected A549 cells collected at 6, 12, 24, and 36 hpi were analyzed by Western blot assay to determine whether ARIH1 expression could be affected by influenza A virus (Fig. 2). The expression of ARIH1 was also detected in HEK293T cells (Additional file 1: Fig. S2). The results indicated that influenza A virus infection increased ARIH1 expression.

### ARIH1 does not affect the internalization and polymerase activity of influenza A virus

**Fig. 3:**
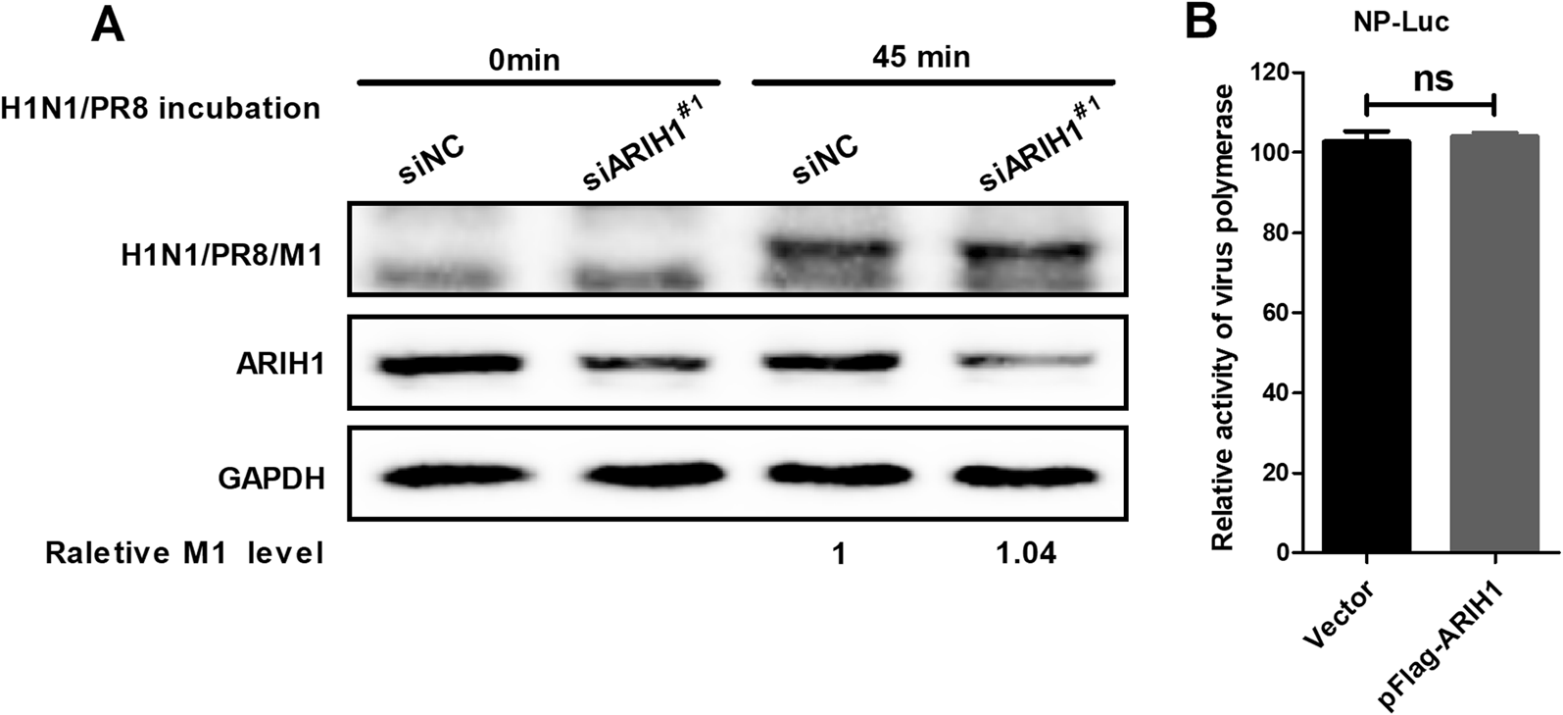
Effect of ARIH1 on influenza A virus internalization and polymerase activity. **A** A549 cells were transfected with siARIH1^#1^ or siNC for 36 h, and then incubated with H1N1/PR8 for 0 or 45 min. Cells were lysed after thorough washing with PBS buffer, and virus internalization was detected by Western blot assay. **B** Polymerase activity was assayed, and each experiment was independently repeated three times. Data are presented as means ± SD from three independent experiments. ns, *P* ≥ 0.05 as determined by student’s *t* test

Virus entry into cells is the first stage to establish infection. Hence, the effect of ARIH1 on the internalization of influenza A virus was examined as previously described [21]. A549 cells were first transfected with siARIH1^#1^ or siNC and then incubated with H1N1/PR8 for 0 min or 45 min at 36 hpi. The cell lysates were collected, and M1 was detected by Western blot assay. No significant change of M1 expression was observed (Fig. 3A). The results showed that ARIH1 did not affect influenza A virus internalization. Polymerase is indispensable for virus replication [22, 23], so luciferase assay was conducted to evaluate polymerase activity. The result showed that ARIH1 did not affect the polymerase activity of influenza A virus (Fig. 3B).

### ARIH1 augments type I interferon

**Fig. 4:**
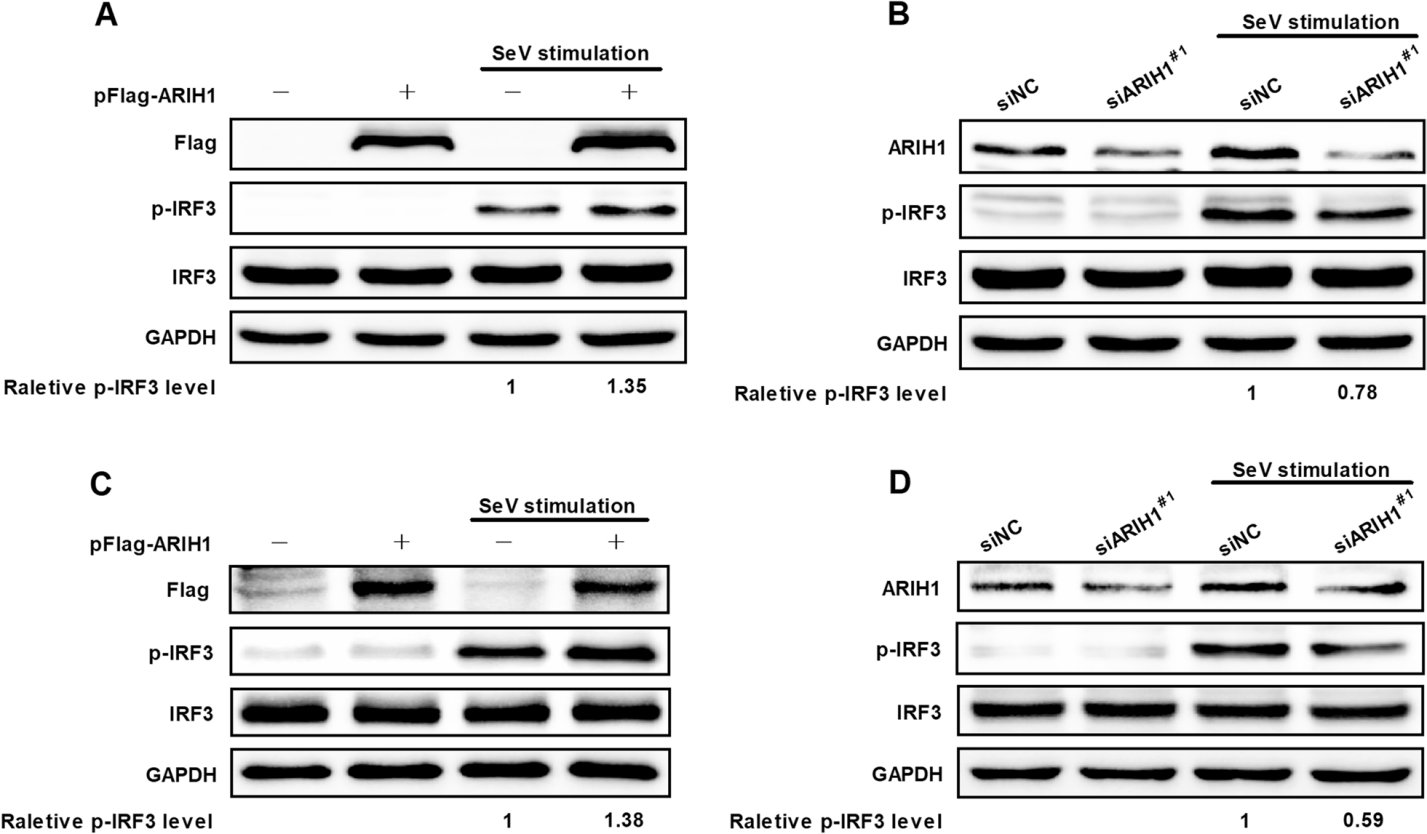
ARIH1 increased phosphorylated IRF3 level. **A** HEK293T cells were stimulated with SeV 24 h after transfection with pFlag-ARIH1 or empty vector. Western blot assay was performed using antibodies specific for Flag, p-IRF3, IRF3, and GAPDH. **B** HEK293T cells were transfected with siARIH1 to silence ARIH1, and siNC was used as control. After 36 h, SeV was used for stimulation, and Western blot assay was performed using antibodies specific for ARIH1, p-IRF3, IRF3, and GAPDH. **C** A549 cells were stimulated with SeV 48 h after transfection with pFlag-ARIH1 or empty vector. Western blot assay was performed using antibodies specific for Flag-tag, p-IRF3, IRF3, and GAPDH. **D** A549 cells were transfected with siARIH1 to silence ARIH1, and siNC was used as control. After 36 h, SeV was used for stimulation, and Western blot assay was performed using antibodies specific for ARIH1, p-IRF3, IRF3, and GAPDH

Type I interferon plays a critical role in innate immune response as the first line of defense against influenza A virus infection [6, 24]. Considering that ARIH1 plays an inhibitory role during H1N1/PR8 infection, we speculated that ARIH1 regulates type I interferon response during virus infection. After ARIH1 was exogenously expressed, p-IRF3 expression increased in HEK293T cells compared with that in vector cells upon SeV stimulation (Fig. 4A). After ARIH1 was endogenously silenced, p-IRF3 expression decreased in HEK293T cells compared with that in siNC-treated cells upon SeV stimulation (Fig. 4B). This finding was consistent with the above results in A549 cells (Fig. 4C, D).

**Fig. 5:**
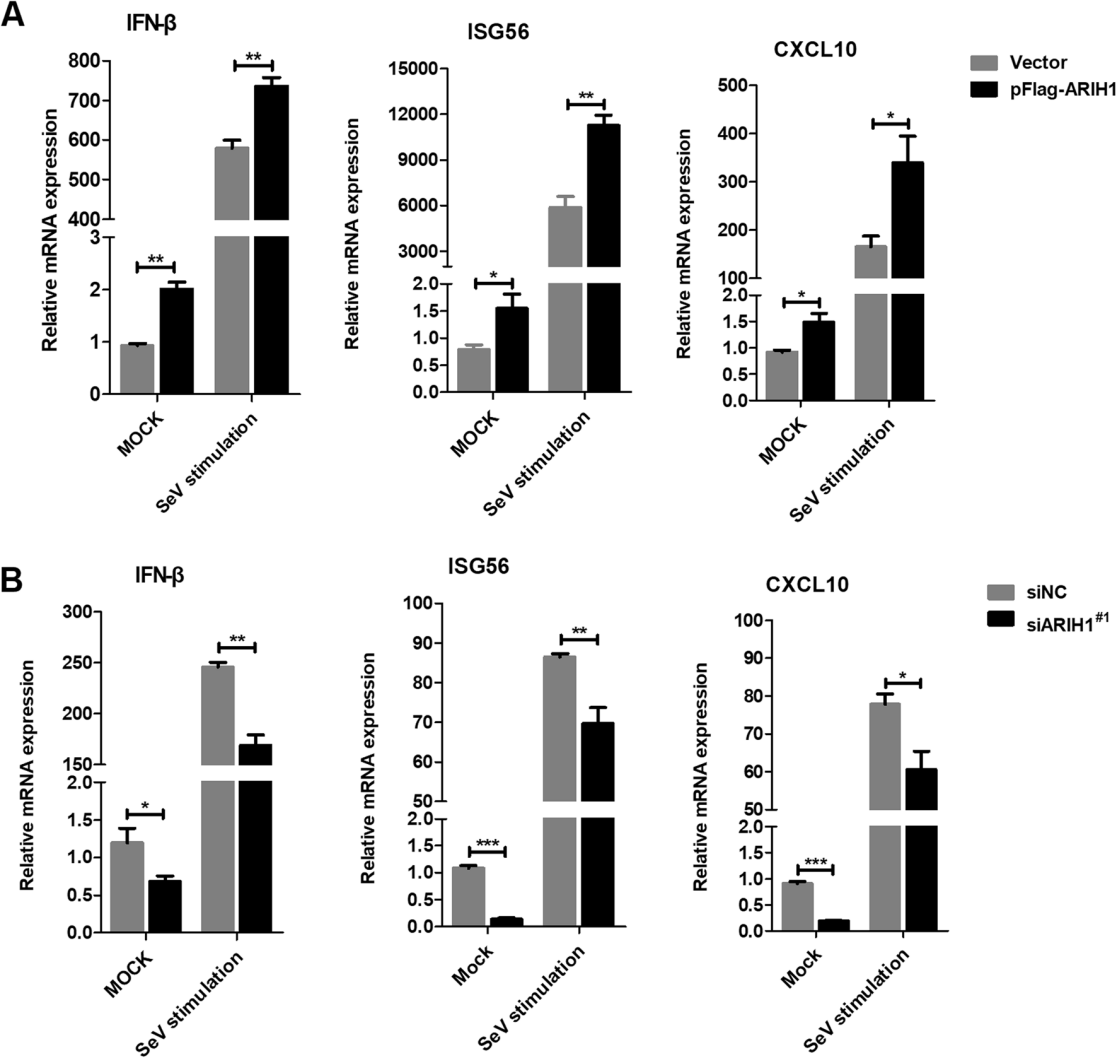
ARIH1 promoted the transcription of IFN-β and its downstream. **A** ARIH1 was overexpressed by pFlag-ARIH1 in HEK293T cells, and the transcription level of IFN-β and its downstream was detected by quantitative RT-PCR assay after stimulation with SeV. **B** ARIH1-silenced HEK293T cells were stimulated with SeV, and the mRNA level of IFN-β and its downstream was tested by quantitative RT-PCR assay. Data are presented as means ± SD from three independent experiments. *, *P* < 0.05 and **, *P* < 0.01, as determined by student’s *t* test

The role of ARIH1 in regulating type I interferon response was also examined. Quantitative RT-PCR assay confirmed that the mRNA level of IFN-β and its downstream increased in the exogenously expressed ARIH1 group during SeV stimulation compared that in the empty vector group in HEK293T cells (Fig. 5A). Tests were also performed after ARIH1 silencing (Fig. 5B). The same results were obtained in A549 cells (Additional file 1: Fig. S3). All these findings corroborated the observation that ARIH1 up-regulats type I interferon response during virus infection.

### ARIH1 indirectly targets RIG-I and does not affect the activation of RIG-I

**Fig. 6:**
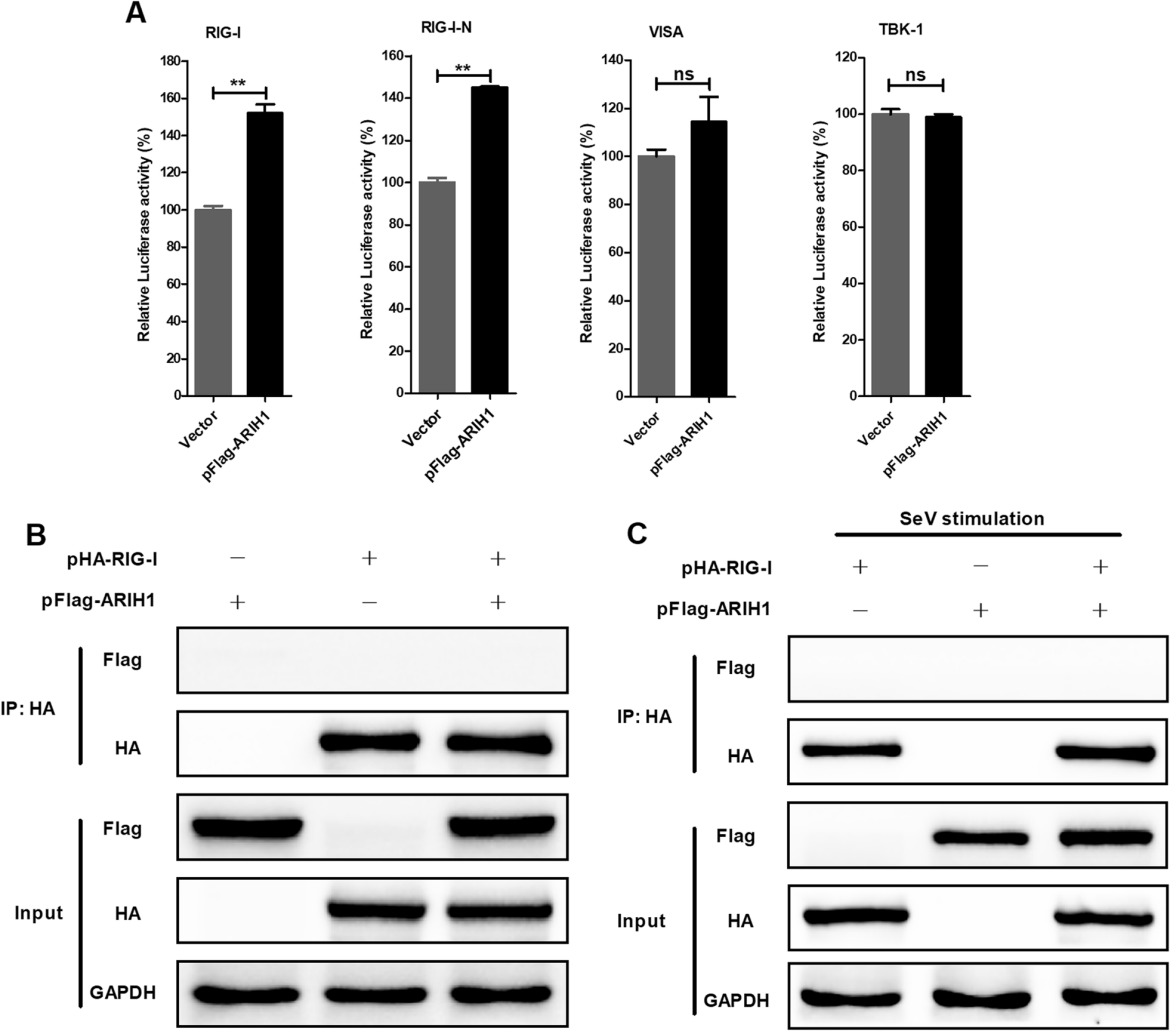
ARIH1 indirectly affected the function of RIG-I. **A** pFlag-ARIH1 or the empty vector was co-transfected with plasmid of RIG-I, RIG-I-N, MAVS, or TBK-1. The luciferase reporter assay was performed. Data are presented as means ± SD from three independent experiments. ns, *P* ≥ 0.05 and **, *P* < 0.01 as determined by student’s *t* test. **B** HEK293T cells were transfected with HA-tag RIG-I plasmid (pHA-RIG-I) and pFlag-ARIH1, and immunoprecipitation assay was performed using HA tag antibody to detect their interaction. **C** HEK293T cells were transfected with pHA-RIG-I and pFlag-ARIH1 and then stimulated with SeV. Immunoprecipitation assay was performed using HA tag antibody to detect their interaction

Influenza A virus infection mainly induces RIG-I signaling pathway [25, 26]. Luciferase reporter assay was performed to determine the target of the inhibitory effect of ARIH1 in the RIG-I signaling cascade. The luciferase activity was enhanced when ARIH1 was co-expressed with RIG-I or RIG-I-N, but MAVS or TBK-1 induced activity was not influenced (Fig. 6A). The interaction between ARIH1 and RIG-I was also detected by immunoprecipitation assay, and coexpression with Flag-tag ARIH1 and HA-tag RIG-I was observed in HEK293T cells. The results revealed the lack of interaction between them with or without SeV stimulation (Fig. 6B, C), confirming that ARIH1 promotes RIG-I-mediated IFN-β promoter activation without directly targeting RIG-I.

**Fig. 7:**
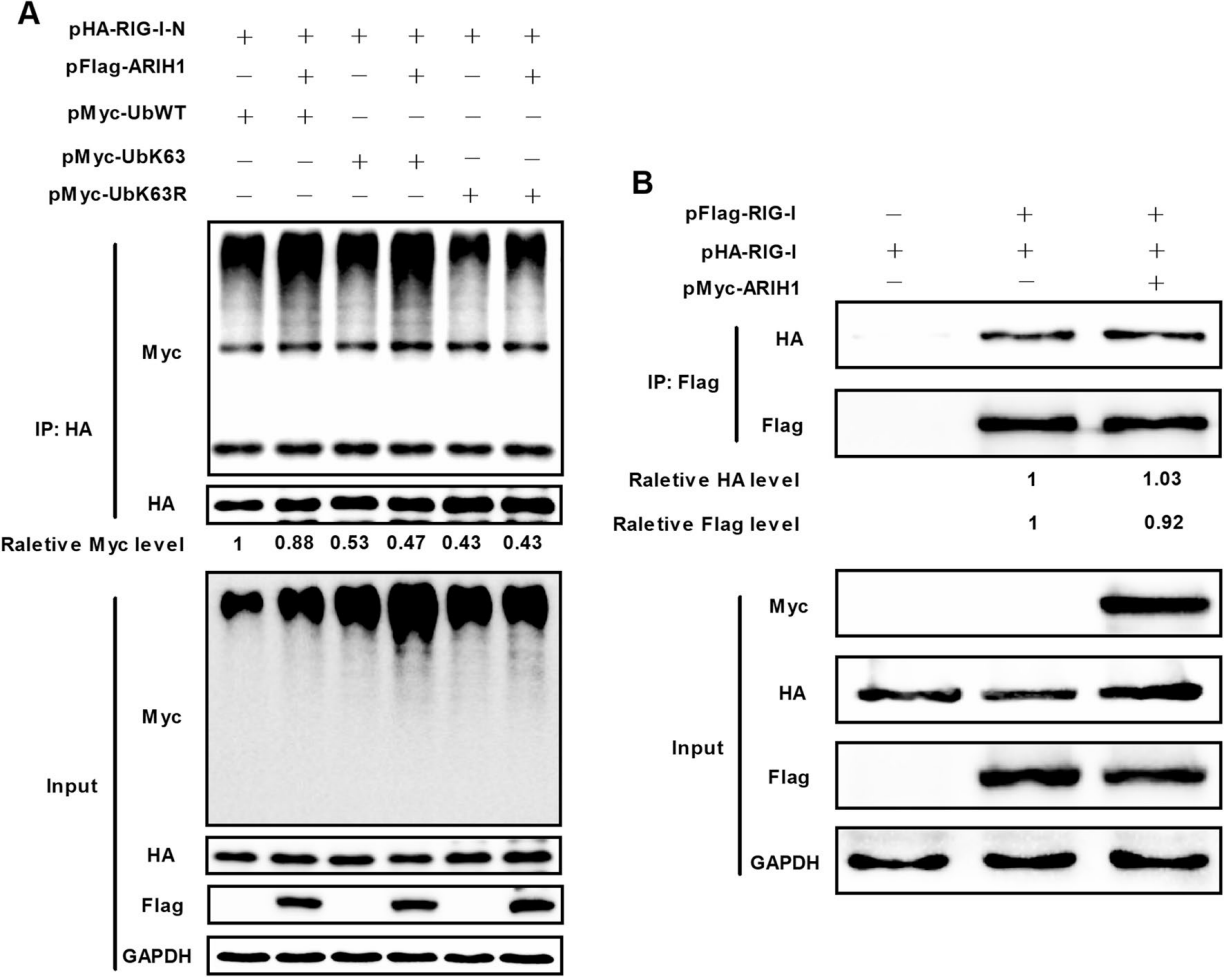
ARIH1 did not affect the K63-linked polyubiquitin and oligomerization of RIG-I. **A** HEK293T cells were co-transfected with pFlag-ARIH1 and HA-tag RIG-I-N plasmid (pHA-RIG-I-N) plus pMyc-UbWT, pMyc-UbK63 or pMyc-UbK63R. Immunoprecipitation assay was performed using HA tag antibody. The polyubiquitination of RIG-I was detected by Myc labeled antibody. **B** HEK293T cells were co-transfected with pHA-RIG-I, FLAG-tag RIG-I plasmid (pFLAG-RIG-I), and Myc-tag ARIH1 plasmid (pMyc-ARIH1) or their corresponding empty vectors, and the effect of ARIH1 on the interaction between HA-tag RIG-I and FLAG-tag RIG-I was detected by immunoprecipitation assay using Flag tag antibody

The K63-linked polyubiquitination of RIG-I is essential for interferon signaling activation, and is regulated by a variety of E3 ubiquitin ligases [13, 27, 28]. HEK293T cells were co-transfected with pFlag-ARIH1 and HA-tag RIG-I-N plasmid (pHA-RIG-I-N) plus Myc-UbWT, Myc-UbK63 or Myc-UbK63R and subjected to immunoprecipitation assay to determine whether the E3 ligase activity of ARIH1 is essential for the K63-linked polyubiquitination of RIG-I. The results showed that ARIH1 did not promote the K63-linked polyubiquitination of RIG-I-N (Fig. 7A). The oligomerization of RIG-I is also critical for RIG-I activation [12, 29, 30]. HEK293T cells were co-transfected with plasmids of pHA-RIG-I, Flag-tag RIG-I plasmid (pFlag-RIG-I), and Myc-tag ARIH1 (pMyc-ARIH1) or their corresponding empty vectors, and the effect of ARIH1 on the interaction between HA-RIG-I and Flag-RIG-I was detected by immunoprecipitation assay. The findings showed that ARIH1 did not affect the interaction between HA-tag RIG-I and Flag-tag RIG-I and consequently the oligomerization of RIG-I (Fig. 7B). All these experiments verified that ARIH1 does not affect the activation of RIG-I.

### ARIH1 interacts with SQSTM1 and upregulates the expression of RIG-I

**Fig. 8:**
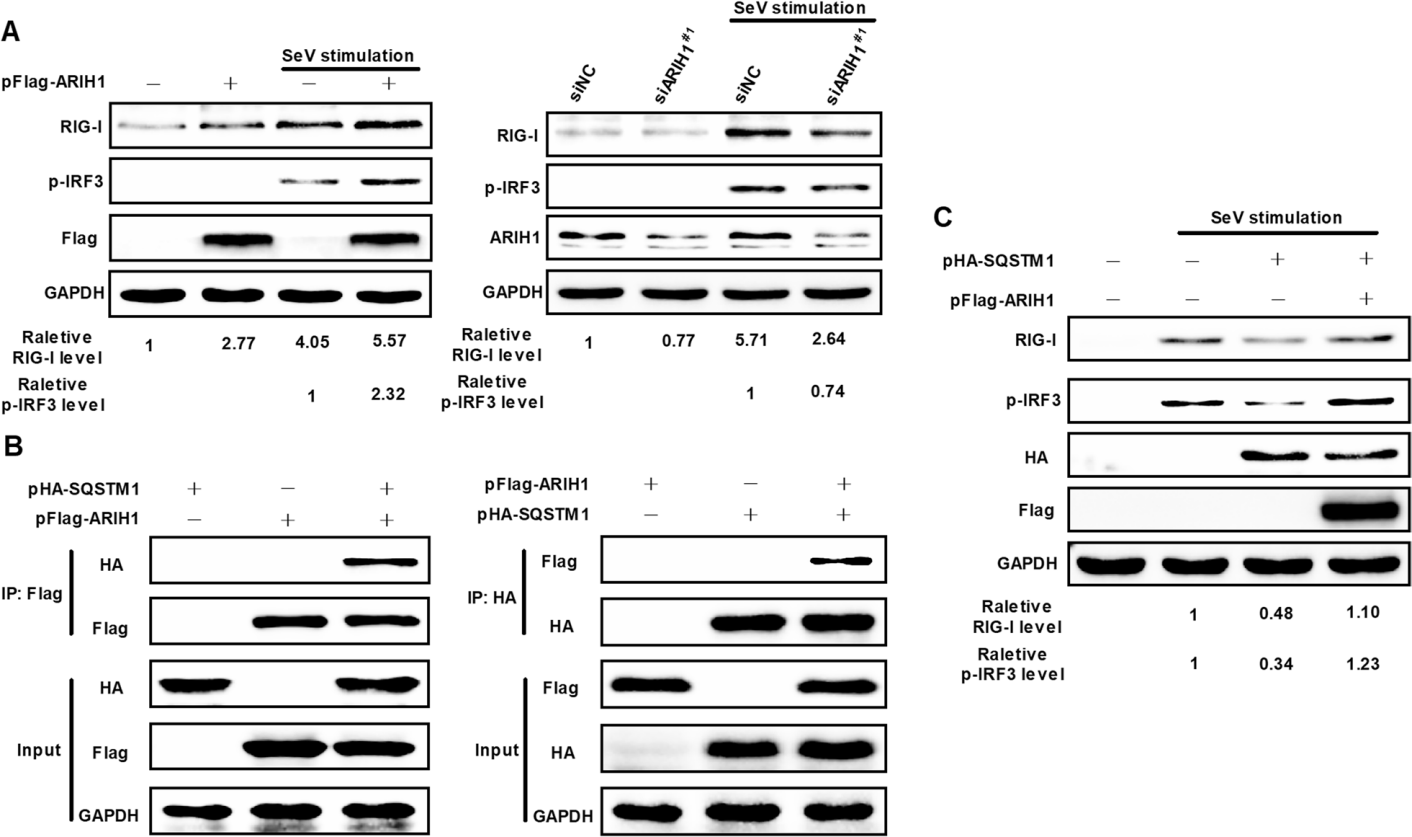
ARIH1 up-regulated the expression of RIG-I by interacting with SQSTM1. **A** ARIH1 was overexpressed or silenced in HEK293T cells, and the expression of endogenous RIG-I was detected by Western blot assay after stimulation with SeV. **B** HEK293T cells were co-transfected with pFlag-ARIH1 and HA-tag SQSTM1 plasmid (pHA-SQSTM1), and immunoprecipitation assay was performed using Flag tag or HA tag antibody to analyze their interaction. **C** HEK293T cells were co-transfected with pHA-SQSTM1 and pFlag-ARIH1 or the corresponding empty vectors and then stimulated with SeV after 24 h. Western blot assay was performed using antibodies specific for RIG-I, p-IRF3, Flag-tag, HA-tag, and GAPDH

RIG-I expression is critical for interferon signaling activation [28, 31, 32]. The expression level of endogenous RIG-I was detected under ARIH1 overexpression and silencing to prove that ARIH1 can affect the expression of RIG-I. The results fully demonstrated that ARIH1 can promote the expression of RIG-I (Fig. 8A). ARIH1 is involved in autophagy regulation, and RIG-I can be degraded by autophagy-related protein SQSTM1/ p62 [32–34]. Whether ARIH1 can affect the function of RIG-I by regulating SQSTM1/ p62 is unknown. Therefore, Flag-tag ARIH1 and HA-tag SQSTM1 were co-expressed in HEK293T cells, and their interaction was detected by immunoprecipitation assay. Western blot assay analysis showed that ARIH1 could interact with SQSTM1 (Fig. 8B). The effect of ARIH1 on the regulation of RIG-I by SQSTM1 was then examined. ARIH1 and SQSTM1 were co-expressed in HEK293T cells and stimulated by SeV after 24 h. The cell lysate was extracted, and the expression of RIG-I and p-IRF3 was detected by Western blot assay. The results showed that ARIH1 could inhibit the degradation of RIG-I by affecting SQSTM1/p62 function (Fig. 8C).

### ARIH1 does not affect polyubiquitination of SQSTM1, but inhibits its binding to RIG-I

**Fig. 9:**
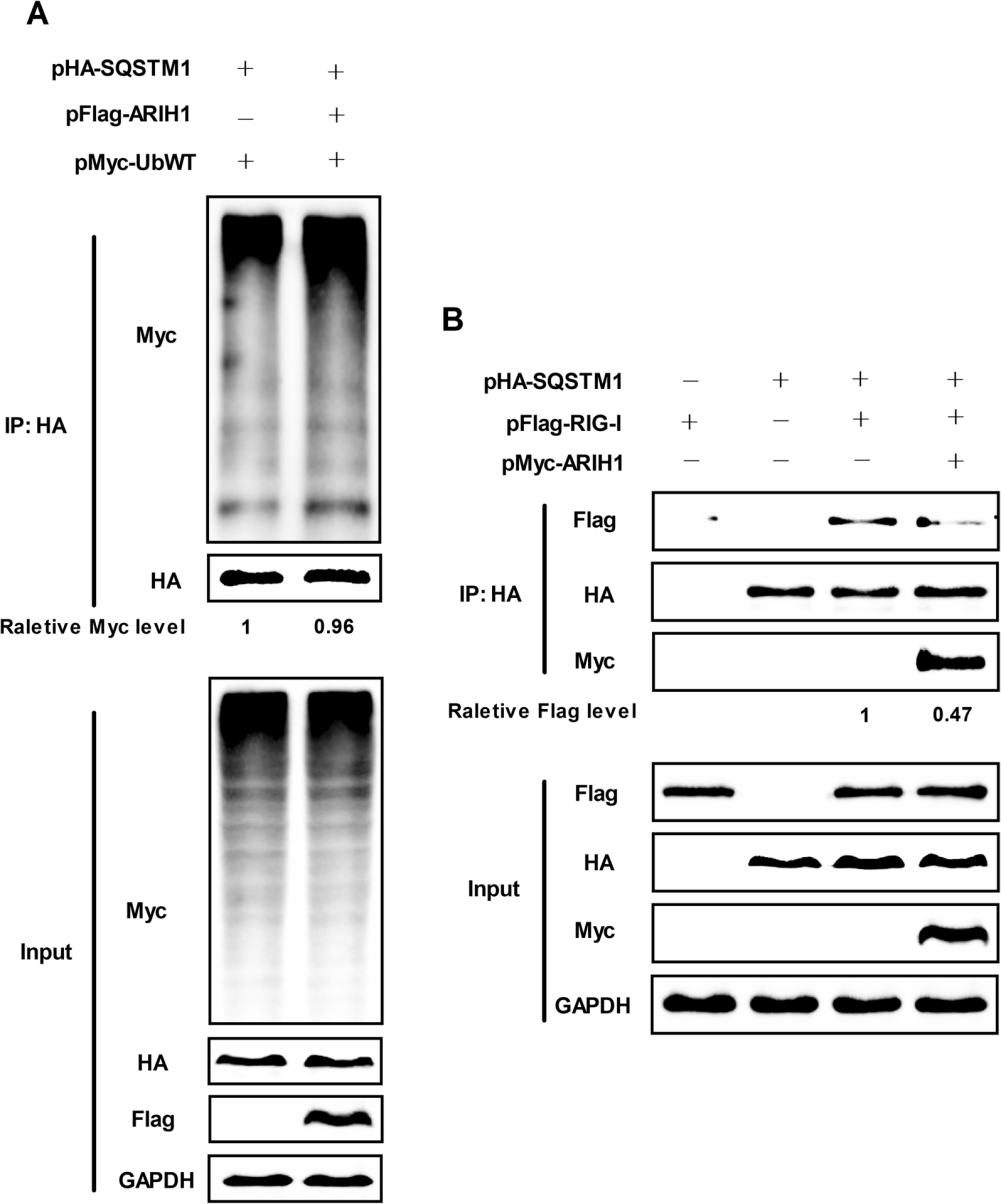
ARIH1 prevented the interaction between SQSTM1 and RIG-I. **A** HEK293T cells were co-transfected with pFlag-ARIH1 or its empty plasmid and pHA-SQSTM1 plus Myc-UbWT, and immunoprecipitation assay was performed using HA tag antibody. The polyubiquitination of SQSTM1 was detected by Myc tag antibody. B HEK293T cells were co-transfected with pFlag-RIG-I and pHA-SQSTM1 plus pFlag-ARIH1 or its empty vector, and the interaction between HA-tag SQSTM1 and FLAG-tag RIG-I was detected by immunoprecipitation assay using HA tag antibody

As E3 ubiquitin ligase, ARIH1 interacts with SQSTM1. We next examined the polyubiquitination of SQSTM1. Immunoprecipitation assay was performed with Flag-tag ARIH1, HA-tag SQSTM1, and Myc-tag UbWT co-expression in cells. The results showed that ARIH1 did not affect the polyubiquitination of SQSTM1 (Fig. 9A). SQSTM1-dependent degradation of proteins pathway plays an important role in signaling pathway regulation [35, 36]. We studied the effect of ARIH1 on the interaction between RIG-I and SQSTM1. Immunoprecipitation assay result showed that ARIH1 impeded the interaction between SQSTM1 and RIG-I (Fig. 9B), proving that ARIH1 promotes the production of interferon by inhibiting the degradation of RIG-I through interference with the interaction between RIG-I and SQSTM1.

## Discussion

ARIH1 belongs to the E3 ubiquitin-protein ligase family, which participates in multiple biological functions. In cancer, ARIH1 can inhibit the function of drugs on tumor cells by regulating autophagy [18, 33] and can act as a suppressor to inhibit tumor development [19]. It also regulates blood glucose, vascular smooth muscle, and iron transport [37–39]. ARIH1 can inhibit the bacterial proliferation and germ cell development of *C. elegans* [40, 41]. However, the antiviral activity of ARIH1 has not yet been sufficiently studied. Our research provides important evidence for this function of ARIH1.

The effect of ARIH1 on the proliferation of influenza A virus was first investigated. The results showed that ARIH1 overexpression inhibited H1N1/PR8 replication, and its silence resulted in the opposite phenomenon (Fig. 1A–D). This study also found that viral infection can up-regulate the expression of ARIH1 (Fig. 2 and Additional file 1: Fig. S2). The effects of ARIH1 on influenza A virus internalization and polymerase activity were examined, but no remarkable influence was found (Fig. 3A, B). This finding suggested that ARIH1 does not affect the proliferation of the virus by affecting its life cycle and implied that viruses may up-regulate ARIH1-induced host antiviral mechanisms. The main antiviral signaling pathway activated by influenza virus in the host is RIG-I signaling pathway, which is commonly stimulated by SeV [13, 26]. Therefore, this study next examined the effect of ARIH1 on SeV-induced phosphorylation of IRF3 in host cells by Western blot assay, which can reflect the activation of the signaling pathways. The results showed that ARIH1 was overexpressed and phosphorylated IRF3 was up-regulated (Fig. 4A, C). Quantitative RT-PCR assay also indicated that ARH1 overexpression increased the mRNA levels of SeV-induced endogenous IFN-β and downstream ISG15 and CXCL10 (Fig. 5A and Additional file 1: Fig. S3A). Opposite results were obtained after the silencing of ARIH1, and the phenomenon was the same in A549 cells and HEK293T cells (Figs. 4C, D, 5A, and Additional file 1: Fig. S3B). The targets of ARIH1 in RIG-I signaling pathway in HEK293T cells were explored using double fluorescence reporting system. Only the overexpression of RIG-I or RIG-I-N enhanced the activation of IFN-β promoter (Fig. 6A). These results indicated that ARIH1 targets at RIG-I and positively regulats the activation of IFN-β signaling.

The activation of RIG-I is the first step for the induction of IFN-β signaling [12, 28, 42]. The interaction between ARIH1 and RIG-I was examined by immunoprecipitation assay, but no relationship was observed (Fig. 6B, C). RIG-I activation involves K63-linked polyubiquitination and oligomerization and various host proteins, such as E3 ubiquitin ligase [27, 29, 43]. As an E3 ubiquitin ligase, the effects of ARIH1 on the K63-linked polyubiquitination and oligomerization of RIG-I were analyzed. The results proved that ARIH1 did not promote the K63-linked polyubiquitination and oligomerization of RIG-I, indicating that ARIH1 does not influence the activation of RIG-I (Fig. 7A, B).

SQSTM1/p62-mediated autophagic degradation is an important mechanism of the negative regulation of type I IFN signaling [32, 44]. ARIH1 is also involved in autophagy [33]. Given that ARIH1 did not affect activation, endogenous RIG-I expression was analyzed by Western blot assay. The results showed that ARIH1 facilitated RIG-I expression (Fig. 8A). Therefore, the effect of ARIH1 on RIG-I regulated by SQSTM1 was then examined. ARIH1 was found to promote RIG-I signaling by inhibiting the degradation of RIG-I through its interaction with SQSTM1 (Fig. 8B, C). Finally, we tested the effect of ARIH1 as E3 ubiquitin ligase on SQSTM1, and discovered that ARIH1 did not affect the ubiquitination of SQSTM1 but could block its binding with RIG-I (Fig. 9A, B). This study provides insights into the underlying mechanism by which ARIH1 promotes IFN-β.

## Conclusions

**Fig. 10:**
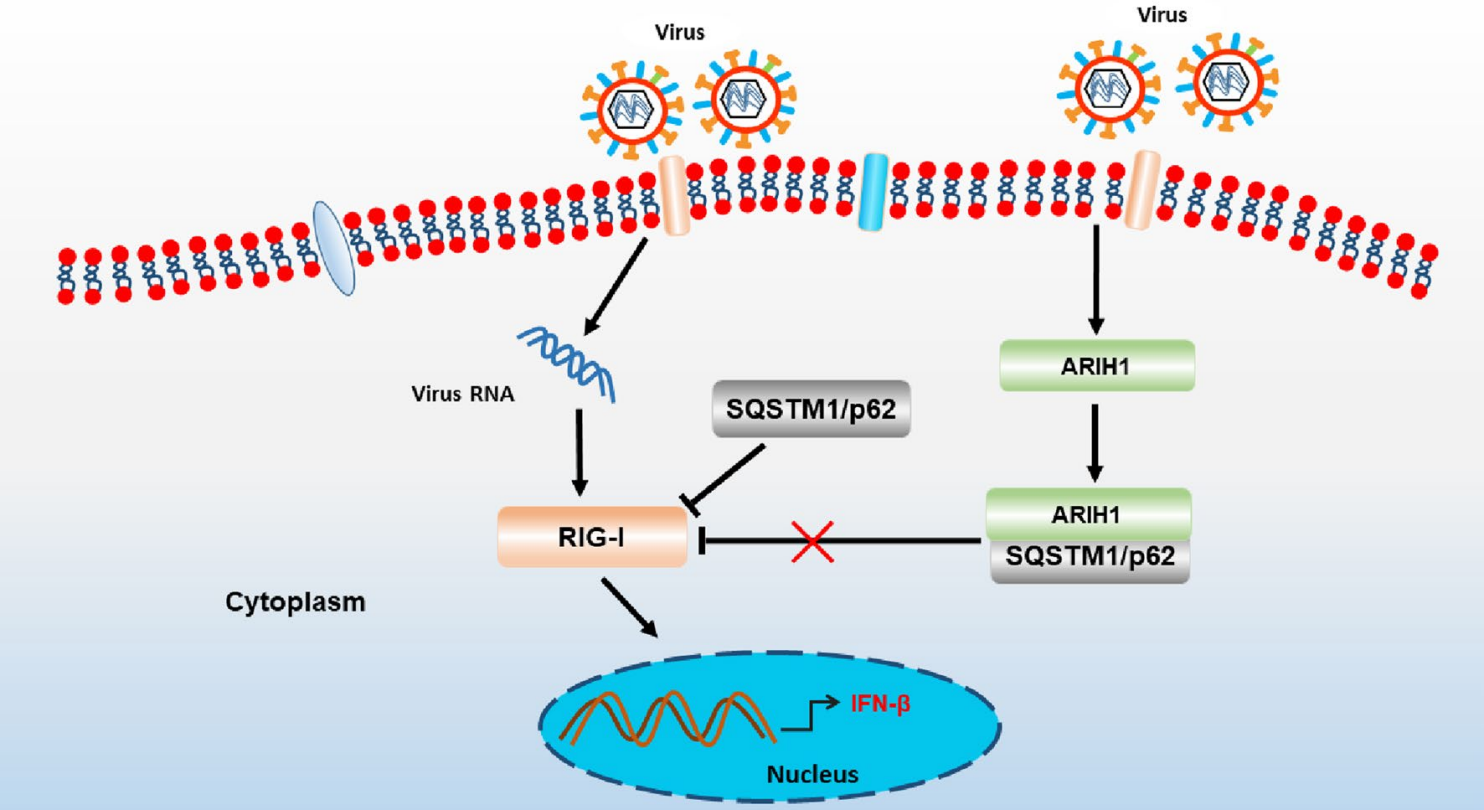
Schematic of ARIH1 action on innate immune signaling pathway. Influenza A virus infection activates RIG-I signaling, which is regulated by a variety of host factors. ARIH1 is up-regulated by influenza A virus and can antagonize the SQSTM1/p62-induced degradation of RIG-I by interacting with SQSTM1 and blocking its binding to RIG-I to promote interferon production

This study proposed a model in which host proteins activate antiviral innate immunity by regulating RIG-I. As a positive regulator, ARIH1 antagonizes the SQSTM1/p62-induced degradation of RIG-I and promotes the activation of IFN-β signaling by interacting with SQSTM1 and blocking its binding to RIG-I. Influenza A virus can up-regulate ARIH1 and promote the expression of IFN-β in infected cells (Fig. 10). However, the mechanism of ARIH1 as an autophagy-related protein that regulate SQSTM1 needs to be further studied.

## Supplementary Information

**Additional file 1. Figure S1.** Screening of siRNA silencing ARIH1. **A** A549 cells were transfected with siARIH1^#1^, siARIH1^#2^ or siARIH1^#3^, and negative control siRNA (siNC) was used as control. After 36h, Western blot assay was performed using antibodies specific for ARIH1 and GAPDH. **B** HEK293T cells were transfected with siARIH1^#1^, siARIH1^#2^ or siARIH1^#3^ for silencing of ARIH1 in cells, and negative control siRNA (siNC) was used as control. After 36h, Western blot assay was performed using antibodies specific for ARIH1 and GAPDH. **Figure S2.** Influenza A virus infection increased endogenous ARIH1 in HEK293T cells. HEK293T cells were infected with H1N1/PR8 for 6 and 12 h. Cell lysates were determined by Western blot assay using antibodies against ARIH1, NP and GAPDH. **Figure S3.** ARIH1 promotes the transcription of IFN-β and its downstream in A549 cells. **A** ARIH1 was overexpressed by pFlag-ARIH1 in A549 cells, and the transcription level of IFN-β and its downstream was detected by quantitative RT-PCR assay after stimulation with SeV. **B** A549 cells silencing ARIH1 by siARIH1^#1^ were stimulated with SeV, and the mRNA level of IFN-β and its downstream was tested by quantitative RT-PCR assay. Data are presented as means ± SD from three independent experiments. ∗∗, *P* < 0.01 as determined by student’s *t* test.

## Data Availability

The datasets used and/or analyzed during the current study are available from the corresponding author on reasonable request.
