# Supplementary material for: ARIH1 inhibits influenza A virus replication and facilitates RIG-I dependent immune signaling by interacting with SQSTM1/p62
Source: Virol J. 2023 Apr 1;20:58. doi: 10.1186/s12985-023-02022-1 (PMC10066941; doi:10.1186/s12985-023-02022-1)
Supplement: Supplementary file 1 — Additional file 1. Figure S1. Screening of siRNA silencing ARIH1. A A549 cells were transfected with siARIH1#1, siARIH1#2 or siARIH1#3, and negative control siRNA (siNC) was used as control. After 36h, Western blot assay was performed using antibodies specific for ARIH1 and GAPDH. B HEK293T cells were transfected with siARIH1#1, siARIH1#2 or siARIH1#3 for silencing of ARIH1 in cells, and negative control siRNA (siNC) was used as control. After 36h, Western blot assay was performed using antibodies specific for ARIH1 and GAPDH. Figure S2. Influenza A virus infection increased endogenous ARIH1 in HEK293T cells. HEK293T cells were infected with H1N1/PR8 for 6 and 12 h. Cell lysates were determined by Western blot assay using antibodies against ARIH1, NP and GAPDH. Figure S3. ARIH1 promotes the transcription of IFN-β and its downstream in A549 cells. A ARIH1 was overexpressed by pFlag-ARIH1 in A549 cells, and the transcription level of IFN-β and its downstream was detected by quantitative RT-PCR assay after stimulation with SeV. B A549 cells silencing ARIH1 by siARIH1#1 were stimulated with SeV, and the mRNA level of IFN-β and its downstream was tested by quantitative RT-PCR assay. Data are presented as means ± SD from three independent experiments. ∗∗, P < 0.01 as determined by student’s t test. [file 12985_2023_2022_MOESM1_ESM.docx]

# Supplementary Figures


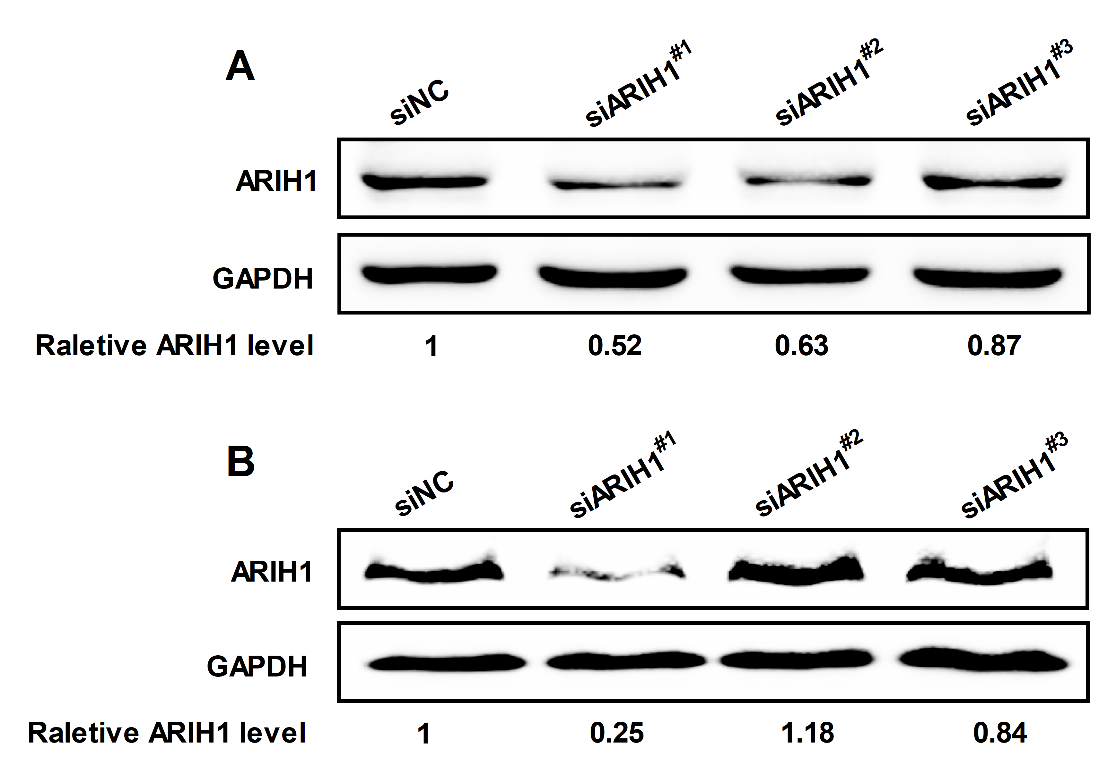


**Fig. S1** Screening of siRNA silencing ARIH1**. A** A549 cells were transfected with siARIH1^#1^, siARIH1^#2^ or siARIH1^#3^, and negative control siRNA (siNC) was used as control. After 36h, Western blot assay was performed using antibodies specific for ARIH1 and GAPDH. **B** HEK293T cells were transfected with siARIH1^#1^, siARIH1^#2^ or siARIH1^#3^ for silencing of ARIH1 in cells, and negative control siRNA (siNC) was used as control. After 36h, Western blot assay was performed using antibodies specific for ARIH1 and GAPDH.


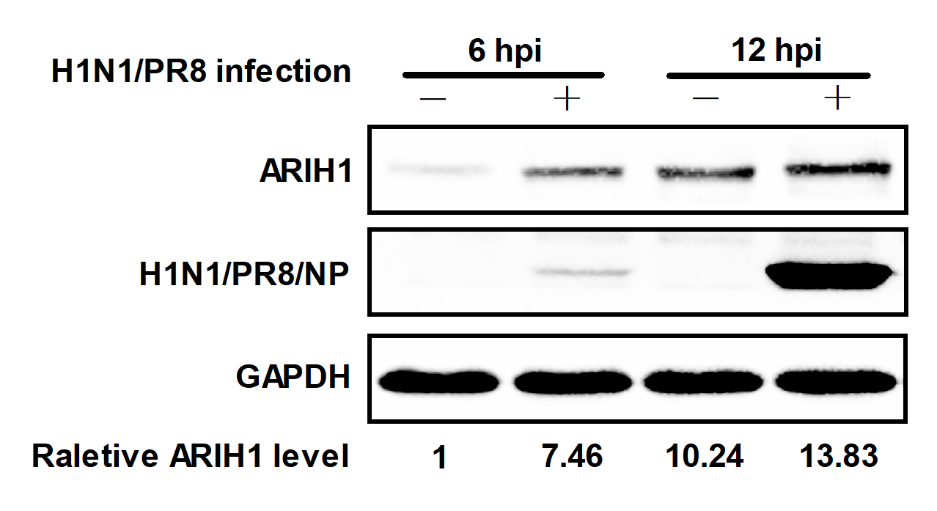


**Fig. S2** Influenza A virus infection increased endogenous ARIH1 in HEK293T cells. HEK293T cells were infected with H1N1/PR8 for 6 and 12 h. Cell lysates were determined by Western blot assay using antibodies against ARIH1, NP and GAPDH.


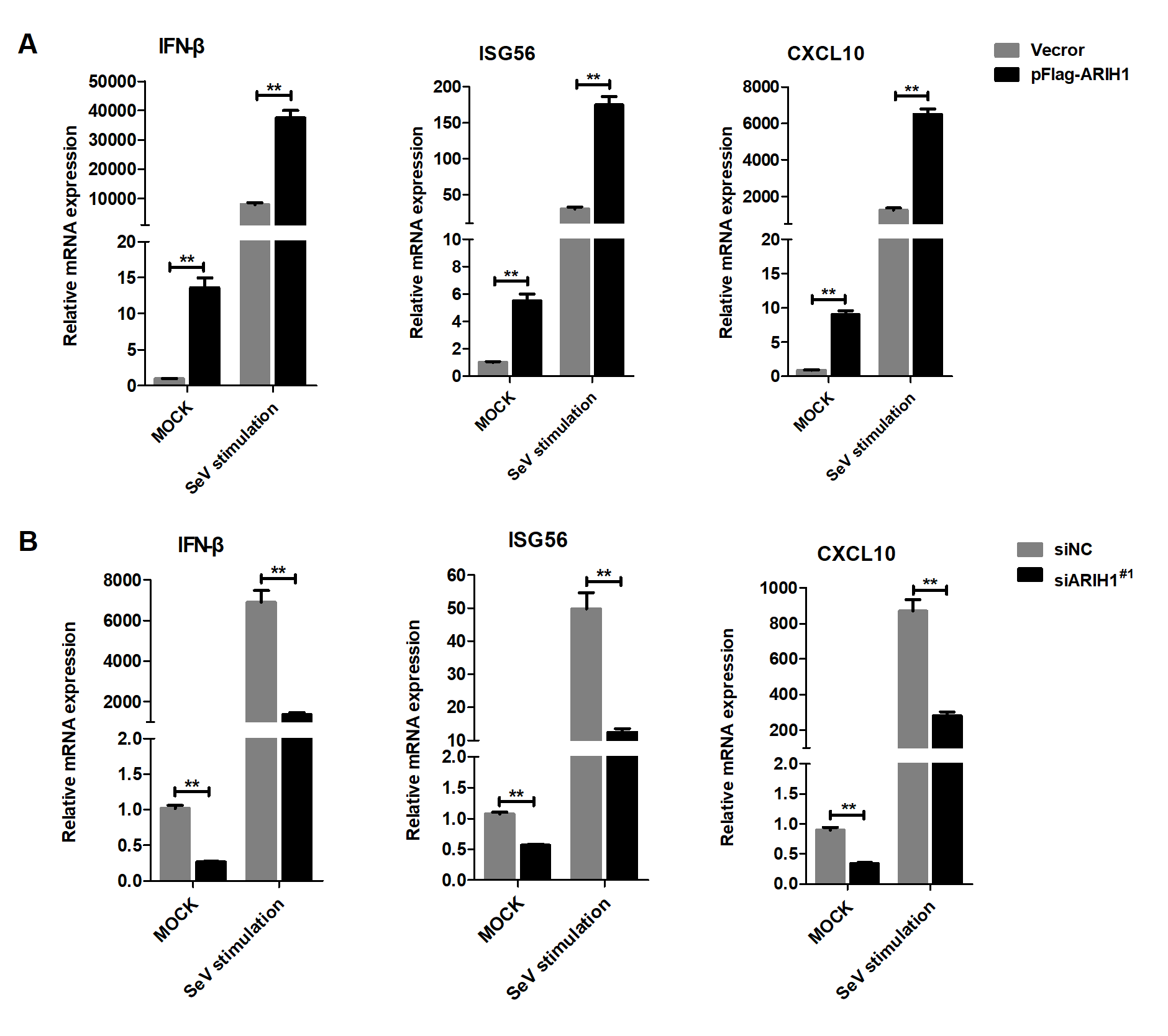


**Fig. S3** ARIH1 promotes the transcription of IFN-β and its downstream in A549 cells. **A** ARIH1 was overexpressed by pFlag-ARIH1 in A549 cells. , and the transcription level of IFN-β and its downstream was detected by quantitative RT-PCR assay after stimulation with SeV. **B** HEK293T cells silencing ARIH1 by siARIH1^#1^ were stimulated with SeV, and the mRNA level of IFN-β and its downstream was tested by quantitative RT-PCR assay. Data are presented as means ± SD from three independent experiments. ∗∗, *P* < 0.01 as determined by student’s *t* test.
